# Deep learning improves physician accuracy in the comprehensive detection of abnormalities on chest X-rays

**DOI:** 10.1038/s41598-024-76608-2

**Published:** 2024-10-24

**Authors:** Pamela G. Anderson, Hannah Tarder-Stoll, Mehmet Alpaslan, Nora Keathley, David L. Levin, Srivas Venkatesh, Elliot Bartel, Serge Sicular, Scott Howell, Robert V. Lindsey, Rebecca M. Jones

**Affiliations:** 1 Imagen Technologies, 224 W 35th St Ste 500, New York, NY 10001 USA; 2 Department of Radiology, Stanford University School of Medicine, 453 Quarry Rd, Palo Alto, CA 94305 USA; 3 The Mount Sinai Hospital, 1 Gustave L. Levy Place, New York, NY 10029 USA

**Keywords:** Radiography, Translational research

## Abstract

Chest X-rays are the most commonly performed medical imaging exam, yet they are often misinterpreted by physicians. Here, we present an FDA-cleared, artificial intelligence (AI) system which uses a deep learning algorithm to assist physicians in the comprehensive detection and localization of abnormalities on chest X-rays. We trained and tested the AI system on a large dataset, assessed generalizability on publicly available data, and evaluated radiologist and non-radiologist physician accuracy when unaided and aided by the AI system. The AI system accurately detected chest X-ray abnormalities (AUC: 0.976, 95% bootstrap CI: 0.975, 0.976) and generalized to a publicly available dataset (AUC: 0.975, 95% bootstrap CI: 0.971, 0.978). Physicians showed significant improvements in detecting abnormalities on chest X-rays when aided by the AI system compared to when unaided (difference in AUC: 0.101, *p* < 0.001). Non-radiologist physicians detected abnormalities on chest X-ray exams as accurately as radiologists when aided by the AI system and were faster at evaluating chest X-rays when aided compared to unaided. Together, these results show that the AI system is accurate and reduces physician errors in chest X-ray evaluation, which highlights the potential of AI systems to improve access to fast, high-quality radiograph interpretation.

**Supplementary Information:**

The online version contains supplementary material available at 10.1038/s41598-024-76608-2.

## Introduction

Chest X-rays are the most commonly performed medical imaging exam, and accurate and timely interpretation of chest X-rays is critical for providing high quality patient care^1^. However, it has been reported that physicians miss findings in approximately 30% of abnormal exams^2,3^, which may lead to delayed treatments, unnecessary costs, malpractice lawsuits, and preventable morbidity^1,4–11^. Physicians, regardless of specialty^12^, can make interpretational errors on radiographs and misinterpretations can arise from many factors including, but not limited to, the number of findings on a radiograph, amount of experience or diagnostic skill of the physician, fatigue, and cognitive or attentional constraints^3,13–21^. Further, the substantially growing workload for radiologists and the need for treating physicians to make time-sensitive decisions results in non-radiologist physicians being tasked with interpreting medical images despite lacking extensive radiology education and training^16,22–25^. Physicians without extensive radiology training are less accurate at interpreting chest X-rays^26–28^ and pose an even larger risk of misdiagnosis^29,30^.

Artificial intelligence (AI) systems using a subclass of AI called deep learning can accurately interpret and classify abnormal findings on chest X-rays^31,32^. These AI models are often highly successful, with performance matching, or even exceeding, that of radiologists^33–40^. However, many of these models have shortcomings, which limit the scope of their clinical applications. Some models only detect select findings on chest X-rays^37–51^ and others have large variations in the amount and quality of data used during training and testing^38^. Additionally, the majority of clinical AI models have not been assessed and cleared through FDA’s rigorous review process^52^. Finally, most AI models have only been shown to improve radiologists’ accuracy^41,53^, but it is important to understand how other physician specialties may benefit from these AI systems because non-radiologist physicians often interpret chest X-rays^22^. For an AI model to be the most beneficial in reducing errors in chest X-ray interpretation, it should be able to detect multiple abnormalities, generalize to new patient populations, and be cleared by FDA to enable clinical adoption across physician specialties^54,55^.

**Figure. 1:**
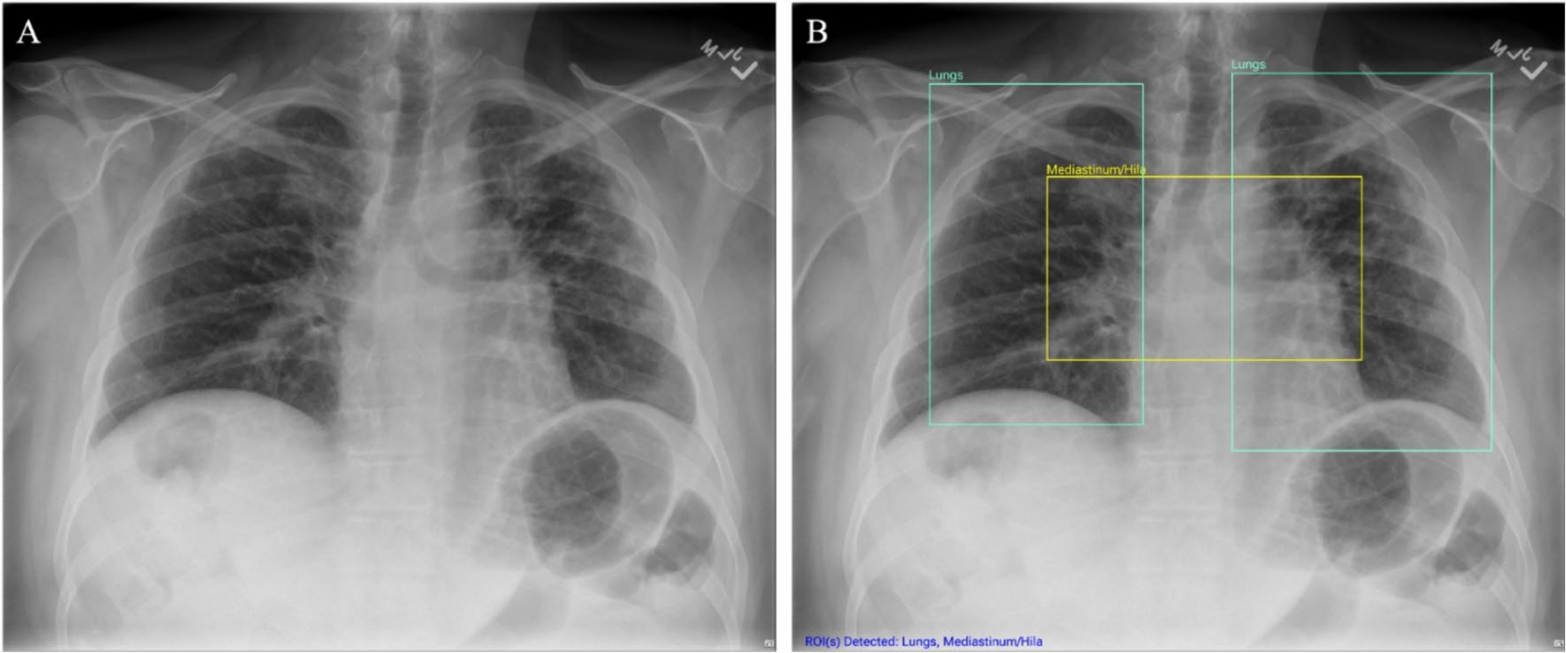
Chest X-ray unaided and aided by the AI system. (**A**) Chest X-ray unaided by the AI system (**B**) Chest X-ray aided by the AI system shows two Lung ROIs (green boxes) and a Mediastinum/Hila ROI (yellow box). The AI system identified abnormalities that were characterized as regions of interest in Lungs and Mediastinum/Hila. The abnormalities were bilateral upper lobe pulmonary fibrosis (categorized as ‘Lungs’), and pulmonary artery hypertension along with bilateral hilar retraction (categorized as ‘Mediastinum/Hila’). The ROIs for each category are illustrated in different colors for readability.

In the current work, we describe our development of an FDA-cleared, computer assisted detection (CAD) system (device name: Chest-CAD, 510(k) number: K210666), which uses deep learning to assist physicians during their interpretation of chest X-rays. The AI system^56^ identifies suspicious regions of interest (ROIs) and assigns each ROI to one of eight clinical categories consistent with the reporting guidelines from the Radiological Society of North America (Cardiac, Mediastinum/Hila, Lungs, Pleura, Bones, Soft Tissues, Hardware, or Other)^57^. The eight categories exhaustively encompass any suspicious ROIs present in a chest X-ray and the AI system produces boxes around the ROIs. Figure 1 shows an example of the AI system output and how the AI system identified three ROIs, and classified them into two categories, Lungs and Mediastinum/Hila. See the Supplementary Information for more details about how the AI algorithm was trained for each category.

To test the efficacy of the AI system, we first assessed performance overall and per category in the absence of any interaction with a physician (referred to as standalone testing) on a dataset of 20,000 chest X-ray cases from 12 healthcare centers in the U.S, which was independent from the dataset used to train the AI system. We also tested the generalizability of our findings on a publicly available chest X-ray dataset. Next, we evaluated radiologist and non-radiologist physician performance with and without the assistance of the AI system (referred to as clinical testing). In this study we aim to find (1) the accuracy of the AI system, trained on a large, diverse dataset (see Table 1 for dataset characteristics), in identifying and categorizing abnormalities on chest X-rays, (2) the consistency of the AI system’s standalone performance across multiple datasets, and (3) the performance of the radiologist and non-radiologist physicians in detecting and categorizing abnormalities on chest X-rays when aided by the AI system compared to when unaided.

## Results

### Evaluation of the AI system

**Figure. 2:**
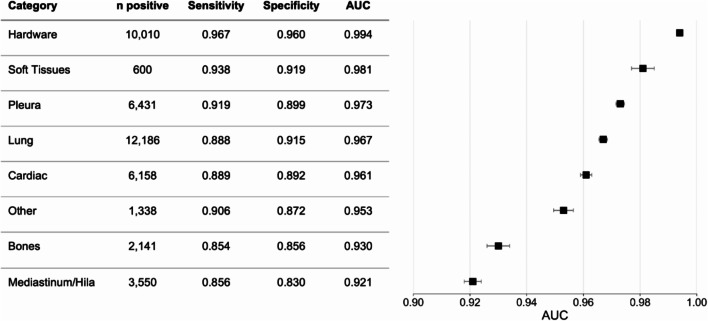
Standalone performance of the AI system per category. Number of positive cases, sensitivity, specificity, and AUC per category in the standalone testing dataset ( n  = 20,000 patient cases). Error bars represent 95% confidence intervals calculated using bootstrap sampling (m = 1000).

We first evaluated the standalone performance of the AI system to determine its accuracy in detecting chest X-ray abnormalities relative to a reference standard defined by a panel of expert radiologists. Details about the reference standard are described in Methods and in Table 1. Standalone performance was assessed on 20,000 adult cases that met the inclusion criteria for the AI system’s indications for use (patient age > = 22 and 1 or 2 image chest X-ray cases). Sampling procedures for the standalone testing dataset are described in the Methods. The AI system had a high level of agreement with expert radiologists and also generalized across patient and image characteristics. The AI system demonstrated high overall AUC (0.976, 95% bootstrap CI: 0.975, 0.976), sensitivity (0.908, 95% bootstrap CI: 0.905, 0.911), and specificity (0.887, 95% bootstrap CI: 0.885, 0.889) in identifying chest abnormalities in the standalone testing dataset. Figure 2 shows the number of positive cases in each of the eight categories, along with high sensitivity, specificity, and AUCs per category. The AI system’s performance was also evaluated across patient and image characteristics in the standalone testing dataset. Supplementary Table 1 shows the AUC by patient sex, patient age group, number of images per case, brightness level, contrast level, resolution level, and X-ray device manufacturer. All AUCs were over 0.950, indicating that the AI system had similarly high performance across subgroups. The accuracy of the ROI location was also compared between the reference standard ROI and the AI system’s ROI by measuring the intersection-over-union (IoU) per category and showed a high degree of overlap^58^ (see Supplementary Table 2).

### Performance of the AI system on the NIH ChestX-ray8 dataset

To test the accuracy of the AI system on an independent chest X-ray dataset, standalone performance was also assessed on a subset of the publicly available National Institutes of Health (NIH) data (ChestX-ray8)^59^. One thousand cases were randomly selected, and analyses were conducted on 922 cases that met the inclusion criteria for the AI system’s indications for use. Details about the reference standard labels are in Table 1 and are described in Methods. The AI system demonstrated high overall AUC (0.975, 95% bootstrap CI: 0.971, 0.978), sensitivity (0.907, 95% bootstrap CI: 0.894, 0.919), and specificity (0.887, 95% bootstrap CI: 0.878, 0.896) in identifying chest abnormalities in the subset of data from the ChestX-ray8 dataset. The AI system also demonstrated high AUCs for each category, consistent with the AUCs in the standalone testing described above, suggesting that the performance of the AI system generalizes to distinct datasets.

### Evaluation of physician performance

**Figure. 3:**
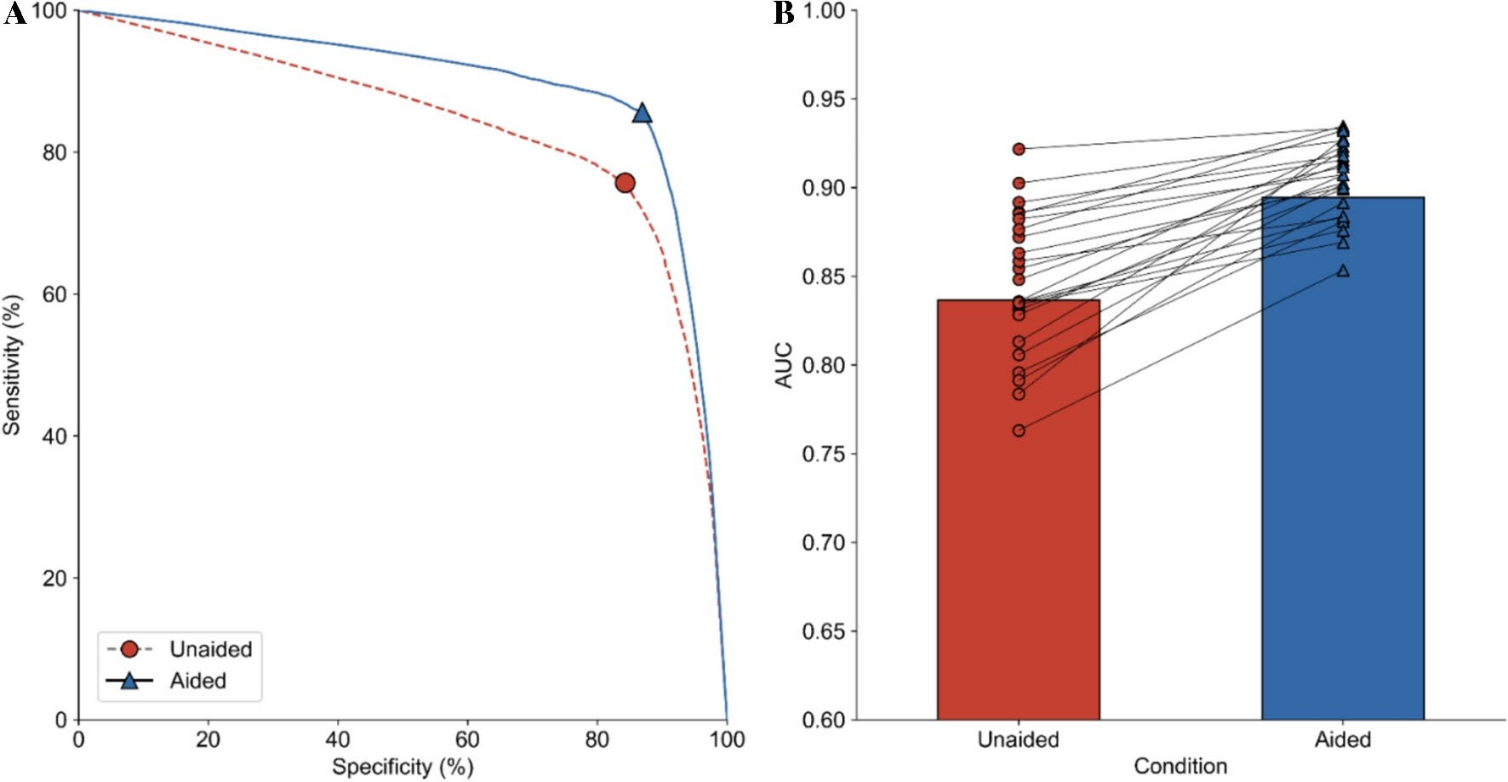
Physician performance unaided and aided by the AI system. A. ROC curve for all physicians when unaided (dashed red line, red circle) and aided (solid blue line, blue triangle) by the AI system. B . AUCs for all physicians when unaided (red bar and circles) and aided (blue bar and triangles) by the AI system. Solid lines represent an individual physician’s improvement when unaided and aided.

We tested physicians’ accuracy in detecting abnormalities on chest X-rays when unaided and aided by the AI system through a multi-reader, multi-case (MRMC) study described in Methods. The AI system significantly improved physicians’ accuracy at detecting chest abnormalities across all categories (*p* < 0.001; unaided AUC: 0.773; aided AUC: 0.874; difference in least squares mean AUC = 0.101, 95% CI: 0.101, 0.102). Figure 3a shows the Receiver Operating Characteristic (ROC) curves when physicians were unaided and aided by the AI system. Physician sensitivity increased from 0.757 (95% CI: 0.750, 0.764) when unaided to 0.856 (95% CI: 0.850, 0.862) when aided, demonstrating that physicians had a relative reduction in missed abnormalities of 40.74%. Physician specificity increased from 0.843 (95% CI: 0.839, 0.847) when unaided to 0.870 (95% CI: 0.866, 0.873) when aided, showing that the use of the AI system did not result in physicians overcalling abnormalities, but instead assisted in correctly identifying cases with no suspicious ROIs. Further, AUC values for every individual physician increased when aided by the AI system compared to when unaided (Fig. 3b; see Supplementary Table 5 for the 24 physicians’ unaided and aided performance).

**Figure. 4:**
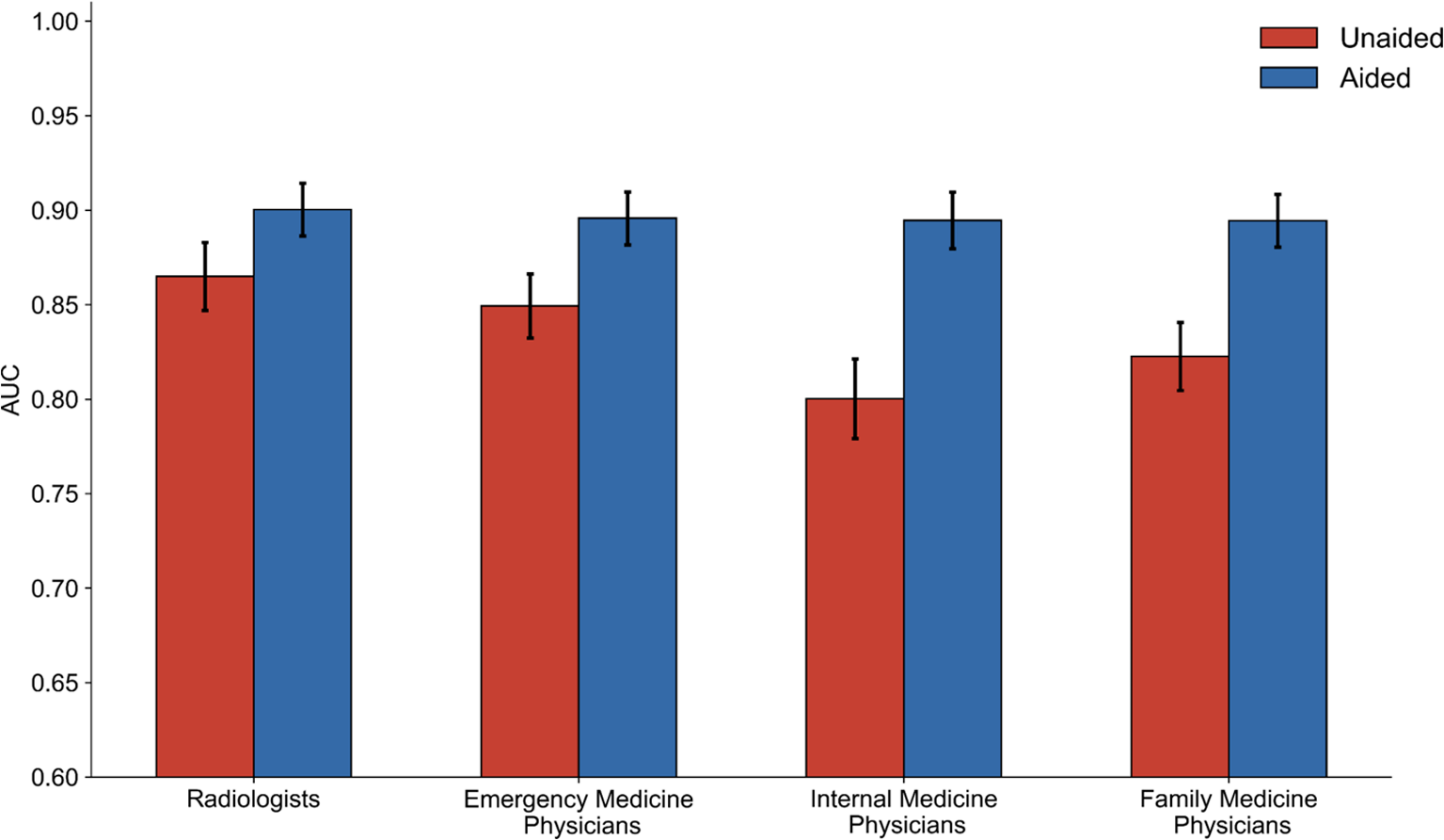
Performance by physician specialty. Average AUC for radiologists, emergency medicine physicians, family medicine physicians, and internal medicine physicians when unaided (red) and aided (blue) by the AI system. Error bars represent 95% confidence intervals calculated using bootstrap sampling (m = 1000).

As expected, radiologists were more accurate than non-radiologist physicians (emergency medicine, family medicine, and internal medicine physicians) at identifying abnormalities in chest X-rays when unaided by the AI system (Fig. 4). Despite high unaided accuracy, radiologists still showed an improvement when assisted by the AI system with an unaided AUC of 0.865 (95% CI: 0.858, 0.872) and an aided AUC of 0.900 (95% CI: 0.895, 0.906). Internal medicine physicians showed the largest improvement when assisted by the AI system with an unaided AUC of 0.800 (95% CI: 0.793, 0.808) and an aided AUC of 0.895 (95% CI: 0.889, 0.900; see Fig. 4). There was a significant difference in AUC between radiologists and non-radiologist physicians in the unaided condition (*p* < 0.001), but no significant difference in the aided condition (*p* = 0.092), suggesting that the AI system aids non-radiologist physicians to detect abnormalities in chest X-rays with similar accuracy to radiologists. Radiologists and non-radiologist physicians also experienced a relative reduction in missed abnormalities of 29.74% and 44.53%, respectively. The AUC values overall and per category for radiologists and non-radiologist physicians when unaided and when aided are reported in Supplementary Table 3.

In addition to physicians demonstrating improved detection accuracy, assistance by the AI system reduced physicians’ case read times. Non-radiologist physicians detected abnormalities in chest X-ray cases significantly faster when aided by the AI system versus when unaided, with an average improvement of 10 s (*t* (1,17) = 2.281, *p* = 0.036 (uncorrected), 7.94% faster, aided median read time = 98.5 s, unaided median read time = 107 s). There was no difference in radiologist read times when aided by the AI system versus when unaided (*t* (1,5) = 0.267, *p* = 0.800, aided median read time = 67.5 s, unaided median read time = 69.5 s; see Supplementary Table 4).

## Discussion

The FDA-cleared AI system demonstrated strong standalone performance, detecting chest abnormalities on X-ray on par with radiologists, and the AI system’s performance generalized to a separate publicly available chest X-ray dataset. In the clinical testing, we showed that overall physician accuracy improved when aided by the AI system, and non-radiologist physicians were as accurate as radiologists in evaluating chest X-rays when aided by the AI system. Taken together, our findings show that the AI system supports different physician specialties in accurate chest X-ray interpretation.

The AI system was trained using deep learning and performed with high accuracy in the standalone testing dataset, with an overall AUC of 0.976. Accuracy remained high across the eight categories of the AI system (all AUCs > 0.92) and across different patient and image characteristics (all AUCs > 0.95). Additionally, we demonstrated the generalizability of the AI system by evaluating performance on the NIH’s publicly available chest X-ray dataset. The system generalized well, with similarly high performance on both datasets (NIH dataset AUC: 0.975; standalone testing dataset AUC: 0.976). The high performance of the AI system can be partly attributed to the large and diverse training dataset and quality of the labels. We manually created over six million labels from nearly 500,000 radiographs. Consistent with our previous work, the labels were from expert U.S. board-certified radiologists to ensure high clinical accuracy and generalizability of the machine learning algorithms^60,61^. Prior work with AI systems for radiography have relied on labels extracted retrospectively from radiology reports using NLP techniques^36,62–64^, which can result in inaccuracies^65–67^. As such, there is a growing consensus that high-quality clinical labels are essential to high-performing AI systems^68–70^.

The AI system has the potential to positively impact patients and the healthcare system as demonstrated by physicians’ consistently higher accuracy at detecting abnormalities on chest X-rays when assisted by the AI system. Every physician in the study improved when aided compared to unaided, suggesting each physician, even those that are highly skilled at chest X-ray interpretation, benefited from using the AI system. There was no difference in accuracy between radiologists and non-radiologist physicians when aided by the AI system. Further, non-radiologist physicians were faster to detect abnormalities on chest X-rays when aided compared to when unaided. This suggests that the AI system increased non-radiologist physicians’ accuracy and efficiency, such that they performed more similarly to radiologists. Improving the accuracy of chest X-ray interpretation with the AI system has the potential to reduce misdiagnosis, leading to improved quality of care and decreased costs, and should be directly measured in future prospective trials.

Improving non-radiologist physicians’ ability to interpret chest X-rays is critical for improving patient outcomes. Non-radiologist physicians’ are often the first to evaluate patients in many care settings and routinely interpret chest X-rays when a radiologist is not readily available^27^. Additionally, the share of practicing radiologists relative to other physician specialties is declining in the U.S.^71^ , particularly in rural counties^71–73^. As a result, practicing radiologists are overburdened and even large institutions, such as the Veterans Affairs hospitals, have backlogs of hundreds of thousands of unread X-ray cases^74^. Therefore, non-radiologist physicians are being tasked with interpreting chest X-rays, despite lack of training and poorer accuracy^22–25^. The majority of prior studies have conducted clinical testing on AI systems with radiologists only^48,50,53^. Here, we provide evidence that the AI system aids non-radiologist physicians, which can lead to greater access to high quality medical imaging interpretation and may reduce misdiagnoses on chest X-rays when radiologists are unavailable.

There were limitations in this study. First, in the clinical study physicians were not given access to additional patient information for the chest X-rays in the study. This was an intentional study design decision to keep the information provided to the physician and the AI system the same. Second, future prospective studies will be necessary to measure the real-world impact of the AI system on directly reducing physicians’ misdiagnoses and subsequently improving patient outcomes. The AI system is well positioned to be implemented and integrated into clinical settings due to its FDA-clearance^56^ and category outputs that align with standard radiology reporting guidelines^57^. Third, a subset of positive cases were labeled for the specific abnormality detected by the expert radiologists’ and the performance of the AI system was evaluated in a more granular manner, however, future analyses with a larger sample size is required to investigate the performance for lower prevalence conditions (see Supplementary Table 6). Finally, while the AI system’s categories were designed to be mutually exclusive, there is a possibility that the expert radiologists interpreted the same abnormality on an X-ray differently and selected two different categories as abnormal when providing ground truth labels. Since the reference standard is determined by the majority opinion, this situation could result in no suspicious ROI being found, which may cause the reported sensitivity values to be higher than the true performance. The expert radiologists received extensive training before providing labels as to the types of abnormalities associated with each category to minimize any confusion about the definitions for each category (see Supplementary Information AI System Categories).

Overall, the FDA-cleared AI system, trained using deep learning, demonstrated strong standalone performance and increased physician accuracy in detecting abnormalities on chest X-rays. It eliminated the gap in accuracy between radiologists and non-radiologist physicians when detecting abnormalities on chest X-rays and facilitated non-radiologist physicians to read cases more efficiently. Thus, the AI system has the potential to increase timely access to high-quality care across the U.S. healthcare system and improve patient outcomes.

## Methods

### AI system development

To build the AI system, 17 U.S. board-certified radiologists with a median of 14 years of experience manually annotated a development dataset of 341,355 chest X-ray cases, generating a total of 6,202,776 labels. The development dataset consisted of 492,996 de-identified radiographs from 185,114 patients collected from 15 hospitals, outpatient care centers, and specialty centers in the United States. The development dataset was then randomly split into a training set (326,493 cases; 471,358 radiographs) and a tuning set (14,862 cases; 21,638 radiographs). See Table 1 for details about the labels, as well as the patient and image characteristics of the development dataset.

**Table 1:** Dataset characteristics for the development, standalone testing, clinical testing, and subset of NIH ChestX-ray8 datasets.

	Development Dataset	Standalone Testing Dataset	Clinical Testing Dataset	Subset of NIH ChestX-ray8 Dataset
Radiographs				
No. of U.S. hospitals, outpatient centers, and specialty centers	15	12	9	n/a
No. of chest cases	341,355	20,000	238	922
No. of radiographs	492,996	28,928	345	922
No. of X-ray device manufacturers	11	9	8	n/a
Patients				
No. of patients	185,114	15,631	236	847
Case Demographics				
No. of female cases (%)	187,488 (54.92)	10,379 (51.90)	132 (55.46)	401 (43.49)
No. 22–44 years old cases (%)	60,180^*^ (17.63)	3,481 (17.41)	29 (12.18)	334 (36.23)
No. 45–64 years old cases (%)	122,307^*^ (35.83)	7,430 (37.15)	92 (38.66)	447 (48.48)
No. 65–74 years old cases (%)	64,365^*^ (18.86)	4,368 (21.84)	53 (22.27)	112 (12.15)
No. 75 + years old cases (%)	90,799^*^ (26.60)	4,721 (23.61)	64 (26.89)	29 (3.15)
Labelers				
No. of U.S. board certified radiologists	17	17	17	13
Median experience (years)	14	14	14	14
No. of total labels	6,202,776	480,000	5,712	22,128
No. of total labels per category	775,347	60,000	714	2,766
No. of positive labels (%)	1,428,303 (23.03)	131,526 (27.40)	1,647 (28.83)	6,617 (29.9)
No. of positive cardiac labels (%)	228,659 (29.49)	18,942 (31.57)	228 (31.93)	462 (16.70)
No. of positive Mediastinum/Hila labels (%)	130,699 (16.86)	12,139 (20.23)	162 (22.69)	664 (24.01)
No. of positive lung labels (%)	394,842 (50.92)	36,639 (61.07)	452 (63.31)	1,979 (71.55)
No. of positive pleura labels (%)	202,697 (26.14)	19,521 (32.54)	230 (32.21)	1,026 (37.09)
No. of positive bone labels (%)	92,514 (11.93)	7,401 (12.34)	103 (14.43)	308 (11.14)
No. of positive soft tissue labels (%)	26,592 (3.43)	2,142 (3.57)	34 (4.76)	204 (7.38)
No. of positive hardware labels (%)	305,516 (39.40)	29,744 (49.57)	360 (50.42)	1,609 (58.17)
No. of positive other labels (%)	46,784 (6.03)	4,998 (8.33)	78 (10.92)	365 (13.20)

* Patient age was missing for 1% of the development dataset due to de-identification procedures so the percentages reported when stratified by age group add up to 99%

To ensure that the radiologists produced high-quality labels for the AI system, each radiologist completed rigorous, multi-step training programs. The expert radiologists were responsible for jointly localizing abnormalities on all radiographs within the case (the localized area was considered the “reference standard” area) and producing case-level judgments as to the presence or absence of abnormalities for each of the eight categories. Each case was interpreted by one to six radiologists. Categories were based on standard radiology reporting guidelines as defined by Radiological Society of North America^57^. The expert radiologists were instructed to look for abnormalities when labeling a suspicious ROI in each category. For example, in the Cardiac category, specific abnormalities the expert radiologists were instructed to look for included cardiomegaly, suspected pericardial effusion, chamber enlargement, valvular calcification, dextrocardia, dextroposition, constrictive pericarditis, coronary artery calcifications, suspected pericardial cyst, and obscured cardiac silhouette. The AI system categories are explained in more depth in Supplementary Information.

The AI system was trained using deep learning to recognize visual patterns that correspond to abnormalities within each category through a supervised training process. The system takes a chest X-ray case as input and produces a binary output indicating the presence or absence of an abnormality for each category. It also produces category-specific bounding box(es) when an abnormal region is identified. The binary outputs for each of the eight categories were defined to be mutually exclusive and collectively exhaustive, so that any clinically relevant abnormality detected by any of the expert radiologists was included in one of the categories.

### Algorithm design

The AI system’s processing of a chest X-ray case consisted of three stages: pre-processing, analysis, and post-processing.

#### Pre-processing stage

Input radiographs from a given chest X-ray case were automatically pre-processed in order to standardize their visual characteristics. Each radiograph, which was required to be a high resolution input (over 1440 pixels), was first cropped to remove excess black padding around the edges. Next, resizing operations that preserved the aspect ratio were applied to standardize image resolution to a height of 800 pixels. The resizing operations downscaled the image with anti-aliasing filters on and with bilinear interpolation. If necessary, in order to reach a target width of 1,024 pixels, the resize operation added padding to the edges of the image. The image was then cropped, if necessary, to a target width of 1,024 pixels.

#### Analysis stage

**Figure. 5:**
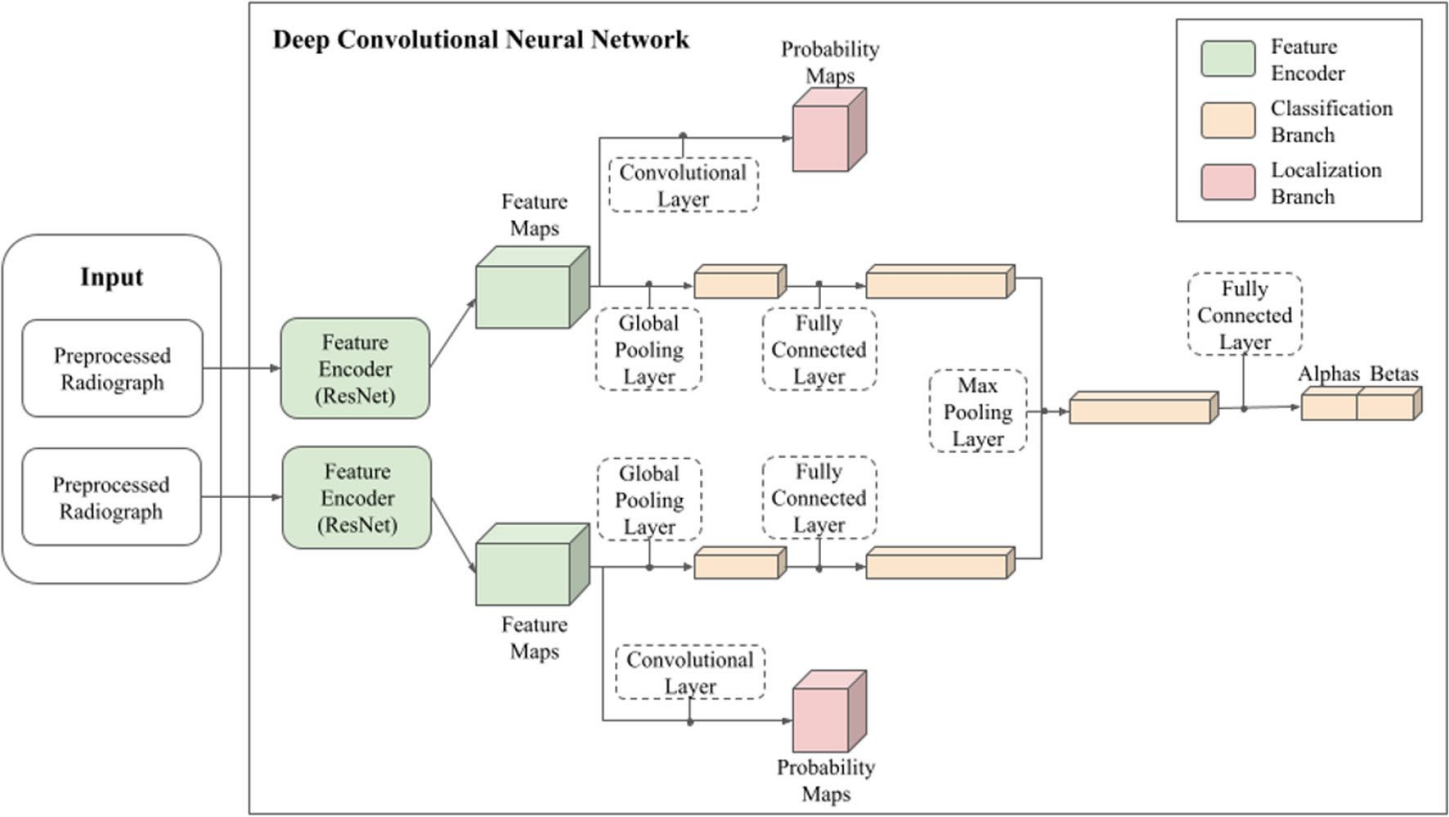
Neural network architecture. The deep convolutional neural network architecture for the AI system is made up of the feature encoder (green), the classification branch (yellow), and the localization branch (red). The feature encoder generates a latent representation of each radiograph. The classification and localization branches use the latent representations of radiographs to produce a probability model of expert radiologists identifying abnormalities for a category and a probability map of where the expert radiologists may identify abnormality for a category, respectively.

The analysis stage took the pre-processed radiographs of a chest X-ray case and used a convolutional neural network to create two outputs per category. One was a Bayesian posterior probability distribution characterizing the model’s belief about the presence of an abnormality within the category. The other was a pixel-wise probability map representing an estimate of where any such abnormality would be within the case. A high-level schematic of the architecture is shown in Fig. 5.

The model produced case-level decisions by jointly analyzing one or more input radiographs from a given case. First, a single case-level embedding was created by separately running each radiograph through an image encoder, producing one 512-dimensional feature vector per radiograph. Each of the 512-dimensional vectors was then passed through a fully connected layer to create 1024-dimensional image feature vectors. Next, the image encodings were collapsed across case-specific radiographs into a single 1024-dimensional feature vector through max-pooling. The image encoder used for this architecture was Resnet-34^75^, which was chosen for parsimony from several options that all yielded similar results (e.g., Densenet). It is a widely used backbone for many computer vision tasks and has a low computational cost compared to many other common architectures. Probability maps for localization were produced by the model through a convolutional layer applied to feature maps from the image encoder’s penultimate layer.

The case embedding was run through a fully connected layer to produce the output parameters “alpha” and “beta” for each category, which parameterized a beta-binomial distribution. From a Bayesian perspective, this distribution represents the model’s prediction for how many *k* expert radiologists out of a group of *n* expert radiologists are likely to identify an abnormality when asked to interpret the chest X-ray. This likelihood model accounts for the observed spread of the available *k*-of-*n* binomial labels, where, as previously described, the binomial labels arose by having multiple expert radiologists review many of the cases within the development dataset. At inference time, a point estimate defined by the mean of the distribution was used by the post-processing stage of the software to determine the presence of a potential abnormality.

#### Post-processing stage

The system took the output from the model and applied post-processing operations to create two outputs per category: (1) a binary determination representing the system’s prediction of whether any abnormalities for that category were present within the X-ray case and (2) a set of bounding boxes surrounding any such abnormalities.

The binary determination was calculated from the point estimates produced by the model using a category-specific threshold pre-computed on the tuning dataset. Any score lying on or above the threshold resulted in an abnormality-present determination, and any score below the threshold resulted in an abnormality-absent determination. The thresholds were optimized to yield equal sensitivity and specificity per category on the tuning set. The set of bounding boxes were created from the category’s pixel-wise probability map output. This was done using a heuristic that placed boxes around the site of high-probability regions using pre-computed, category-specific thresholds that binarized the probability map.

#### Model training

The model was trained by minimizing a joint loss function that assessed the model’s ability to correctly predict the case-level classifications (“ROI present” or “ROI absent” for each output category) and the ability to correctly predict the location of the abnormality within individual radiographs. The joint-loss function was defined as a weighted sum of two terms for each category and then summed across categories. The first term was the across-radiograph average per-pixel binary cross-entropy loss between a given radiograph’s predicted probability map and the ground truth map for that radiograph. The second term was the negative log-likelihood of the beta-binomial distribution, where the distribution’s two free parameters (denoted as “alpha” and “beta” in Fig. 5) were outputs of the model. The binomial observations used in the beta-binomial negative log-likelihood correspond to *k* out of *n* labeling radiologists indicating the presence of an abnormality for the given case and category; note that *n* and *k* vary per case, where some cases were labeled by only a single expert radiologist and others were labeled by multiple independent expert radiologists. To increase the robustness of the model, data augmentation was used during training. Radiographs were randomly rotated, vertically or horizontally flipped, gamma-corrected, contrast-adjusted, and cropped.

The training algorithm repeatedly iterated through the training set in randomized batches of 64 cases. The parameters of the model were updated after processing each batch to minimize the aforementioned loss function. This minimization was achieved using a variant of the stochastic gradient descent algorithm called AdamW^76^. After each epoch, the model was evaluated on the tuning set. An early stopping criterion (across-category mean AUC on the tuning set) was used to determine the continuation of model training based on whether an improvement occurred in the last 10 epochs.

After training finished, the chest X-ray cases in the tuning set were run through the trained model. Prediction scores on cases in the tuning set were used to compute the operating point for each category in the final model. The resulting decision thresholds (one per category) were then fixed and held constant prior to testing.

### Standalone evaluation

#### Standalone testing dataset

**Figure. 6:**
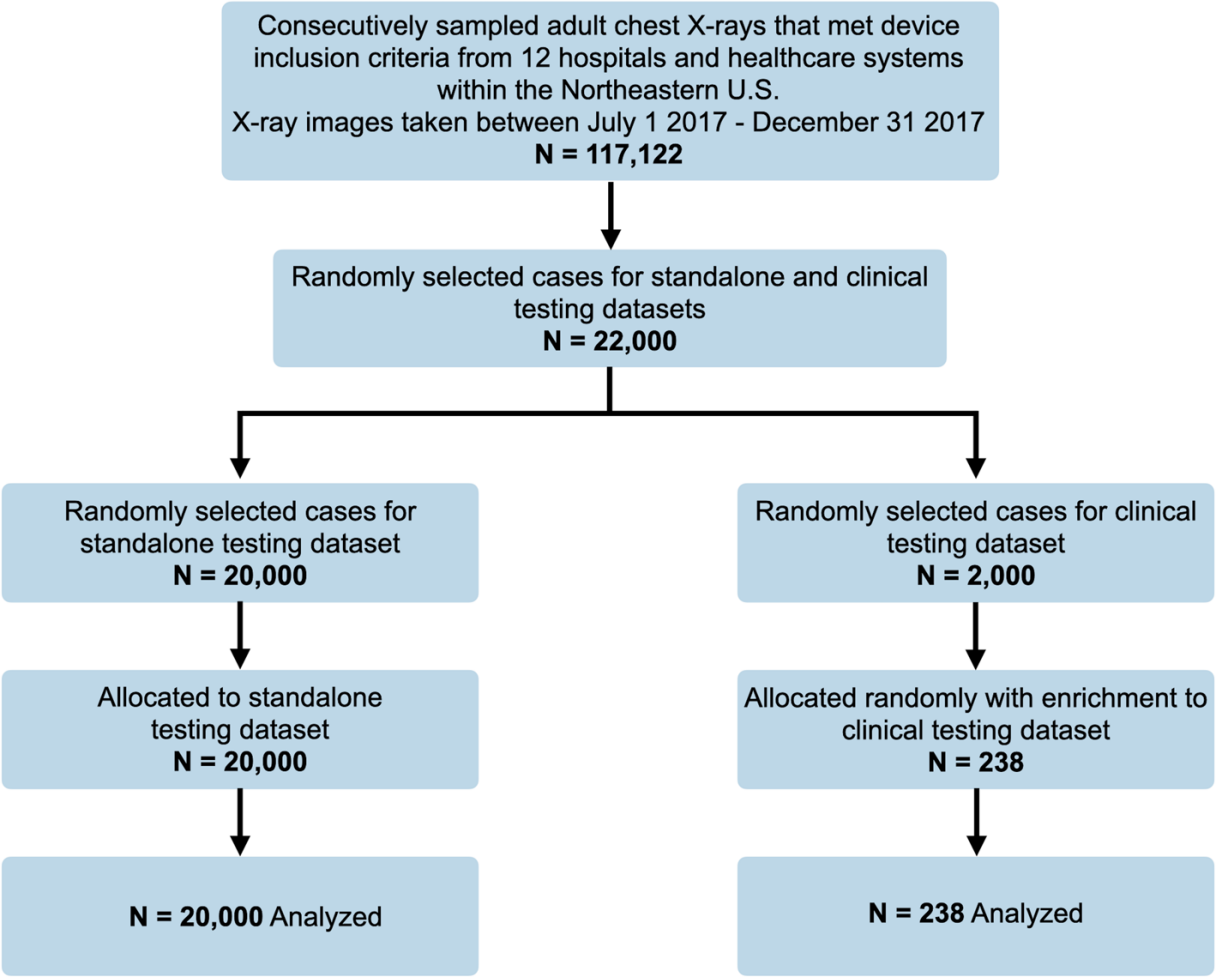
Chest X-ray sampling procedures. Flow diagram illustrating chest X-ray sampling procedures for the standalone and clinical testing datasets.

The AI system’s performance was evaluated on a standalone testing dataset consistent with FDA guidelines^77^. The standalone testing dataset consisted of 20,000 chest X-ray cases that were retrospectively randomly sampled from 12 hospitals and healthcare centers between July and December of 2017 to create a set of patient cases that was representative of the AI system’s intended use (see Fig. 6). The 12 hospitals and healthcare centers were a subset of the 15 hospitals and healthcare centers that were used for the training dataset. There was a diligent segmentation process to minimize patient overlap between the testing and training sets. There was an overlap of 0.33% of the patients in the training dataset with the patients in the standalone testing dataset, however, the impact of these overlapping patients was negligible on the study results as the performance remained unchanged when these patients were removed from the analysis (overall AUC remained 0.976). Reference standard labels for the standalone dataset were provided by 17 U.S. board-certified radiologists with a median of 14 years of experience post-residency. Each case was independently reviewed by three expert radiologists, and if any findings associated with the category (see Supplementary Information AI System Categories) was identified then the expert radiologist would determine a positive ROI for that category. The reference standard for each category was determined by the majority opinion (at least two of three) of the reviewing expert radiologists. During labeling, each expert radiologist was instructed to make a binary decision on the presence or absence of an abnormality for each of the categories, for each chest X-ray case they were assigned. Twenty thousand cases ensured a large natural positive incidence rate for each category with at least 600 reference standard positive cases per category. See Table 1 for the number of positive cases per category, details about the labels used to create the reference standard, and the patient and image characteristics for the standalone testing dataset. See Fig. 6 for data sampling procedures for the testing datasets. There was no case overlap between the development dataset and the standalone testing dataset.

Performance of the AI system was evaluated using the area under the curve (AUC) of the receiving operator characteristic (ROC) curve per category and overall. The “overall” calculations were performed by aggregating the data from all categories and all cases through a technique called micro-averaging^78^. The sensitivity and specificity were also calculated per category and overall. The 95% confidence intervals were reported for each metric using bootstrap resampling (m = 1000).

To measure the localization accuracy of the AI system’s ROIs, the Intersection-over-Union (IoU) was used to calculate the overlap of the predicted bounding box(es) and the reference standard bounding box(es) for each category. When multiple predicted bounding boxes were present for a category, the areas were summed to find the total predicted area. Similarly, when multiple reference standard bounding boxes were found for a category, the areas were also summed to find the total reference standard area. The IoU is determined by finding the area of the intersection of the predicted area with the reference standard area and dividing by the union of the predicted area with the reference standard area. The reported aggregate IoU per category is the average IoU of the true positive cases.

To measure the performance of the AI system on identifying category-specific ROIs when particular abnormalities are present on chest X-rays, labels were collected from the U.S. board-certified radiologists on a subset of 400 to 800 positive cases per category as determined by the reference standard. The abnormality labels were collected on a random subset of positive cases, therefore, not all abnormalities were represented in the investigation and only those found on over 20 cases were analyzed. The volume of cases with each type of abnormality identified can be found in Supplementary Table 6 along with the AI system’s sensitivity in detecting the corresponding category-specific ROI.

#### NIH ChestX-ray8 dataset

The AI system performance was evaluated on a subset of cases from the ChestX-ray8 dataset from the National Institutes of Health to confirm the robustness of the AI system. The complete ChestX-ray8 dataset is made up of over 100,000 de-identified chest X-rays obtained from over 30,000 patients^59^. We randomly sampled 1,000 cases and 78 cases were removed because they did not meet the inclusion criteria for the AI system’s indications for use. Thirteen U.S. board certified radiologists with a median of 14 years post-residency labeled each of the 922 cases for the presence or absence of suspicious ROIs for each of the eight categories. We established the reference standard using majority opinion by randomly selecting labels from three of the U.S. board certified radiologists. See Table 1 for details about the labels, as well as the patient and image characteristics of the NIH dataset. The sensitivity, specificity, and AUC of the ROC curve was calculated overall and per category.

### Physician evaluation

To evaluate whether physician accuracy improved when aided compared to unaided by the AI system, we conducted a multi-reader, multi-case (MRMC) study consistent with FDA guidelines^79^. Twenty-four physicians were enrolled to represent the intended use population. The physician specialties included radiologists (*n* = 6), internal medicine physicians (*n* = 6), family medicine physicians (*n* = 6), and emergency medicine physicians (*n* = 6). Physicians had a mean 14.9 years of experience (range: 1–38 years).

Each physician read 238 X-ray cases (190 cases with at least one abnormality and 48 cases with no abnormalities) unaided and aided by the AI system. Physicians were not given access to additional patient information beyond the chest X-ray. The chest X-ray cases used in the clinical testing dataset were retrospectively randomly sampled from 12 hospitals and healthcare centers between July and December of 2017. Enrichment procedures ensured that there were at least 10 positive cases for each category in the clinical testing dataset. See Table 1 for details about the labels used to create the reference standard, as well as the patient and image characteristics for the clinical testing dataset. The reference standard labels were developed with the same methods outlined in the standalone testing dataset section above. See Fig. 6 for data sampling procedures for the clinical testing dataset. There was no case overlap between the development dataset, the standalone testing dataset, and the clinical testing dataset.

A power analysis was conducted in advance of the study, and it determined that using 24 physicians and 238 cases would provide over 90% power to detect a difference in aided versus unaided AUCs of 0.04. The study consisted of two independent reading sessions separated by a washout period of at least 28 days to reduce memory bias. Physicians read all cases twice. In the first session, half the cases were aided by the AI system and the other half were unaided. In the second session, all cases were read in the opposite condition. Cases were assigned using randomized stratification to reduce case order effects. Each physician was asked to determine the presence or absence of an abnormal region of interest for each of the eight categories for every case and provide a confidence score (0–100) for each of their judgments.

To determine whether there was a statistically significant difference between physician performance when aided versus unaided, we used the Dorfman, Berbaum, and Metz (DBM) model. The DBM model is one of the most common methods for estimating differences in the area under the curve (AUC) of the receiver operating characteristic (ROC) curve and for calculating the corresponding confidence intervals for MRMC studies^80,81^. We also calculated the sensitivity and specificity for each physician type, as well as 95% bootstrap confidence intervals. In addition, we calculated the relative reduction in miss rate rate by subtracting physicians’ aided miss rate (1 - sensitivity) from their unaided miss rate (1 - sensitivity), and then dividing by their unaided miss rate (1 - sensitivity).

To examine the impact of the AI system on physicians who specialize in medical image interpretation versus those who do not specialize in medical image interpretation, we separated the physicians into two groups, radiologists and non-radiologist physicians (internal medicine, family medicine, and emergency medicine physicians). The “overall” AUCs for radiologists and non-radiologist physicians were found by aggregating the data per physician group from all categories and all cases by micro-averaging^78^. To evaluate the performance between radiologists and non-radiologist physicians AUCs in the unaided and aided setting, we performed the Delong test^82^ and evaluated the p-values against a Bonferroni-adjusted alpha level of 0.025 (0.05/2). We again calculated the relative reduction in miss rate for radiologists and non-radiologist physicians, respectively, by subtracting each group’s aided miss rate (1- sensitivity) from their unaided miss rate (1 - sensitivity), and then dividing by their unaided miss rate (1 - sensitivity). To evaluate if the AI system significantly improved case read times, we first took the log transform of the read times to create a normal distribution. Then, we performed a paired t-test to compare log read times in the unaided and aided settings for radiologists and non-radiologist physicians, respectively.

### Ethics approval

IRB approval was obtained from the New England IRB (study #1283806).

## Supplementary information

Below is the link to the electronic supplementary material.

Supplementary Material 1

## Data Availability

The 1,000 randomly selected cases from the NIH ChestX-ray8 dataset and the corresponding labels are available upon reasonable request. The output of the model and the reference standard labels used to calculate the standalone results in this study are available upon reasonable request. Access to the X-ray images used in the standalone and physician evaluation are not publicly available under Imagen Technologies’ license.

## Declarations

### HIPAA compliance

All Protected Health Information used in the training and validation of this deep learning system was de-identified in compliance with the Healthcare Information Portability and Accountability Act of 1996 (HIPAA)’s Expert Determination method. The study complied with all relevant ethical regulations, and a patient waiver of consent was granted by the New England Independent Review Board because the study presented no risk to patients.
