# Supplementary material for: Deep learning improves physician accuracy in the comprehensive detection of abnormalities on chest X-rays
Source: Sci Rep. 2024 Oct 24;14:25151. doi: 10.1038/s41598-024-76608-2 (PMC11502915; doi:10.1038/s41598-024-76608-2)
Supplement: Supplementary file 1 — Supplementary Material 1 [file 41598_2024_76608_MOESM1_ESM.docx]

# Supplementary Information

## AI System Categories

The eight categories of the AI system are based on the Radiological Society of North America reporting guidelines[^5^](https://www.zotero.org/google-docs/?7TWFYl)^7^. Information about how the AI algorithm was developed for each category is described below. To further illustrate the categories for the AI system, we provide a list of abnormalities that the expert radiologists were instructed to look for when labeling a suspicious ROI within each category. These labels were used to train the underlying algorithm.

**Cardiac**: The Cardiac category was developed using radiographs with abnormalities of the heart and pericardium. Specific abnormalities the expert radiologists were instructed to look for included cardiomegaly, suspected pericardial effusion, chamber enlargement, valvular calcification, dextrocardia, dextroposition, constrictive pericarditis, coronary artery calcifications, suspected pericardial cyst, and obscured cardiac silhouette (e.g., total hemithorax opacification).

**Mediastinum/Hila**: The Mediastinum/Hila category was developed using radiographs with abnormalities of the aorta, pulmonary artery, trachea, esophagus, mediastinal lymph nodes, and the hilar regions adjacent to the heart. Specific abnormalities the expert radiologists were instructed to look for included severe tracheal calcification, severely tortuous aorta, severe aortic calcification, aortic aneurysm, right sided aortic arch, mediastinal mass or lymphadenopathy, tracheal pathology (e.g., deviation due to underlying mass effect, enlarged paratracheal stripe, tracheal narrowing), pneumomediastinum, prominent main pulmonary artery segment, calcified mediastinal nodes, esophageal pathology (e.g., achalasia, colonic interposition after esophagectomy, stomach pull-up after esophagectomy, hiatal hernia), mediastinal shift, mediastinal widening, obscured mediastinum (e.g., total hemithorax opacification), enlarged hilar vessels in the setting of pulmonary edema or pulmonary hypertension, hilar retraction (e.g., in the setting of significant pulmonary volume loss), hilar lymphadenopathy or mass, calcified hilar nodes, and obscured hila (e.g., total hemithorax opacification or a large pleural effusion).

**Lungs**: The Lungs category was developed using radiographs with abnormalities within the lungs and included abnormal opacity and abnormal aeration. Specific abnormalities the expert radiologists were instructed to look for included azygos fissure or other similar congenital lung abnormality, pulmonary nodules, any abnormal lung opacity (e.g., pneumonia, consolidation, mass, interstitial lung disease, cephalization, congestion, interstitial edema, pulmonary alveolar edema, hemorrhage, ARDS, bronchiectasis, juxtaphrenic peaking, fibrosis, significant atelectasis/scarring, bullous disease/blebs), hyperinflated lungs, decreased lung volumes, calcified granulomas, and silhouetted diaphragm.

**Pleura**: The Pleura category was developed using radiographs with abnormalities of the entire pleural space. Specific abnormalities the expert radiologists were instructed to look for included pneumothorax, apical pleural capping or other opacity, pleural scarring, pleural effusion, pleural plaques, pleural mass, and indeterminate cases (e.g., collimated or obscured costophrenic angles).

**Bones**: The Bones category was developed using radiographs with any abnormalities of visible bones (e.g., the ribs, spine, clavicles, shoulders). Specific abnormalities the expert radiologists were instructed to look for included severe degenerative changes, Diffuse Idiopathic Skeletal Hyperostosis (DISH), severe arthritis of any joint, severe rotator cuff disease, surgical changes to a bone, moderate or severe Scoliosis or Kyphosis, fractures of any age, dislocation and subluxation, bone lesions, severe osteopenia, diffuse sclerosis, renal osteodystrophy, osteolysis, loose bodies (synovial chondromatosis), and congenital anomalies (e.g., pectus excavatum, pectus carinatum, cervical rib, vertebral segmentation anomalies).

**Soft Tissues**: The Soft Tissues category was developed using radiographs with abnormalities of the soft tissues of the chest wall, back, and shoulders. Specific abnormalities the expert radiologists were instructed to look for included heterotopic ossification, chondrocalcinosis, notable breast findings (e.g., breast implants, mastectomy), calcific tendonosis, non-specific soft tissue calcification when significant, soft tissue emphysema, joint effusion, cachexia, and Poland syndrome.

**Hardware**: The Hardware category was developed using radiographs with iatrogenic objects that were internal to the patient, such as lines, tubes, and hardware, regardless of whether they were normal or abnormal in position. Specific abnormalities the expert radiologists were instructed to look for included pacemaker, ICD, generator device, PICC, CVC, Port-a-Cath, IABP, Feeding tube, Gastrostomy tube, drainage catheter, chest tube, tracheostomy, endotracheal tube, vascular stent, IVC filter, ventriculoperitoneal shunt catheter, spinal stimulator, defibrillator pad, surgical clips and material (e.g., CABG, cholecystectomy, thyroidectomy, lobectomy sutures, breast biopsy clips, bone cement, skin staples, etc.), nipple piercing, nipple marker, musculoskeletal fixation hardware, sternal wires, cardiac prosthetic valves, cardiac closure devices, coronary stents, casts, and foreign body (e.g., shrapnel, ingested foreign body).

**Other**: The Other category was developed using radiographs with abnormalities of 4 areas: diaphragm, abdomen, neck, and peripheral vasculature. Specific abnormalities the expert radiologists were instructed to looked for included diaphragmatic eventration, renal or enteric contrast, abnormal stomach distention, abnormal bowel distention, pneumoperitoneum, portal venous gas, pneumobilia, gallstones, porcelain gallbladder, emphysematous cholecystitis, renal stones, pancreatic calcifications, ascites, peripheral vascular disease involving the extremities, carotid artery, and renal artery, abdominal aortic aneurysm, splenic artery aneurysm, and neck pathology (e.g., neck mass).

## Supplementary Figures and Tables

**Supplementary Figure 1: Performance of the AI system per category on the NIH dataset.**

Number of positive cases, sensitivity, specificity, and AUC per category in the subset of NIH ChestX-ray8 dataset. Error bars represent 95% confidence intervals calculated using bootstrap sampling (m=1000).

**
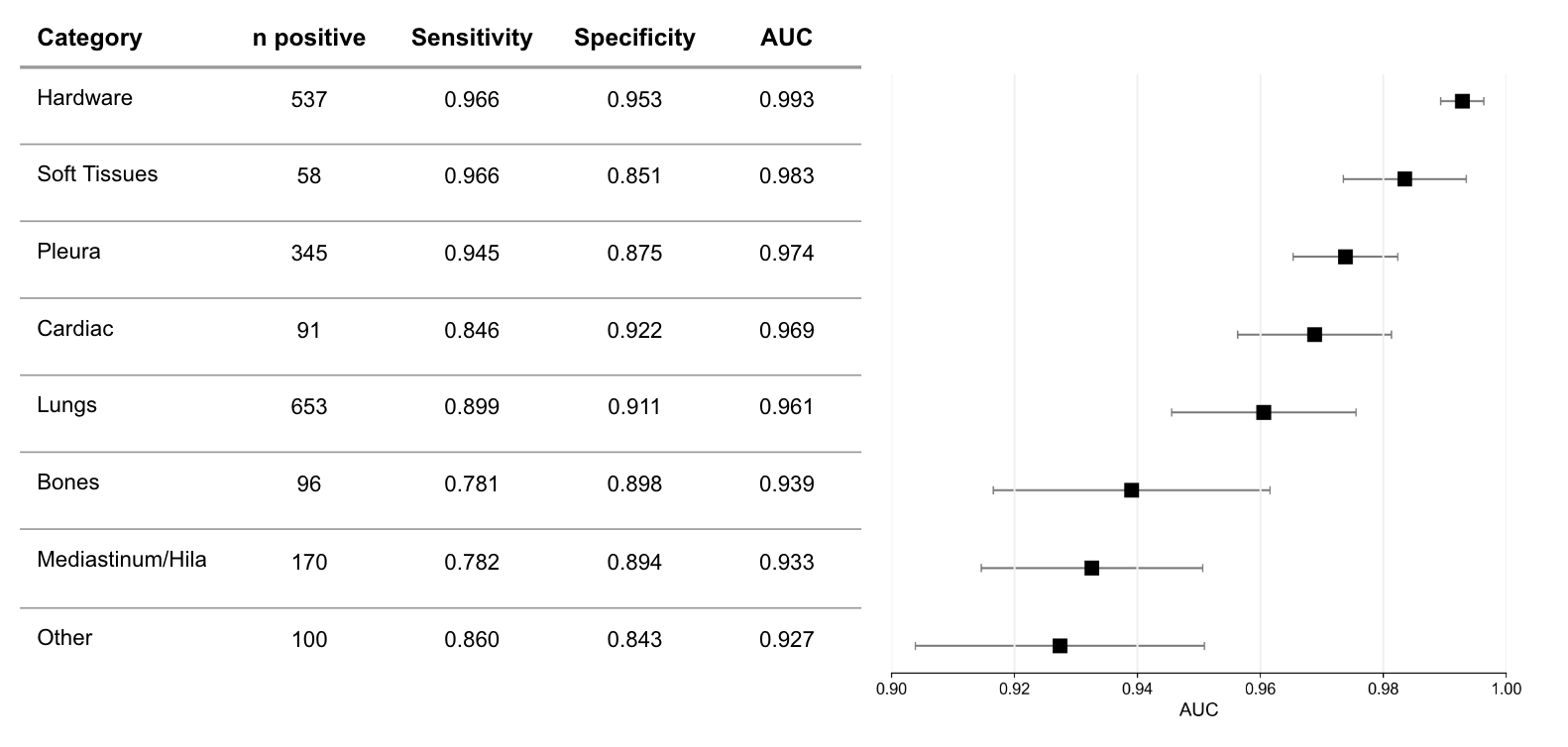
**

**Supplementary Table 1: Performance of the AI system across patient subgroups and image characteristics in the standalone dataset.**

|  | **n** | **AUC** | **95% Bootstrap CI** |
| --- | --- | --- | --- |
| **Patient Sex** | | | |
| Male | 9,621 | 0.976 | 0.975, 0.977 |
| Female | 10,379 | 0.975 | 0.975, 0.976 |
| **Patient Age Group** | | | |
| 22-44 | 3,481 | 0.986 | 0.984, 0.987 |
| 45-64 | 7,430 | 0.979 | 0.978, 0.980 |
| 65-74 | 4,368 | 0.971 | 0.970, 0.972 |
| 75+ | 4,721 | 0.962 | 0.961, 0.964 |
| **Number of Images Per Case** | | | |
| 1 | 11,072 | 0.973 | 0.972, 0.974 |
| 2 | 8,928 | 0.978 | 0.977, 0.979 |
| **Brightness** | | | |
| Low | 6,666 | 0.977 | 0.976, 0.979 |
| Medium | 6,668 | 0.976 | 0.975, 0.978 |
| High | 6,666 | 0.972 | 0.970, 0.973 |
| **Contrast** | | | |
| Low | 6,670 | 0.975 | 0.974, 0.977 |
| Medium | 6,664 | 0.977 | 0.976, 0.978 |
| High | 6,666 | 0.974 | 0.973, 0.975 |
| **Resolution** | | | |
| Low | 7,870 | 0.974 | 0.973, 0.975 |
| Medium | 5,462 | 0.974 | 0.972, 0.975 |
| High | 6,668 | 0.978 | 0.977, 0.979 |
| **Manufacturer*** | | | |
| Carestream Health | 1,479 | 0.975 | 0.973, 0.977 |
| Fujifilm Corporation | 2,794 | 0.971 | 0.970, 0.973 |
| GE Healthcare | 1,721 | 0.976 | 0.974, 0.978 |
| Kodak | 1,968 | 0.977 | 0.975, 0.978 |
| Konica Minolta | 58 | 0.974 | 0.962, 0.987 |
| Philips Medical Systems | 2,129 | 0.980 | 0.979, 0.982 |
| Samsung Electronics | 6,454 | 0.972 | 0.971, 0.973 |
| Siemens | 3,396 | 0.978 | 0.977, 0.980 |

* The Manufacturer Agfa was not part of the analysis due to sparse frequency.

**Supplementary Table 2: Intersection-over-Union statistics for the AI system per category.**

| **Category** | **Intersection-over-Union (IoU)** |
| --- | --- |
| Cardiac | 0.717 |
| Hardware | 0.580 |
| Mediastinum/Hila | 0.518 |
| Lungs | 0.480 |
| Other | 0.479 |
| Pleura | 0.364 |
| Soft Tissues | 0.360 |
| Bones | 0.332 |

**Supplementary Table 3: Accuracy gap between radiologists and non-radiologist physicians when unaided and aided by the AI system.**

| **Category** | **Unaided Performance** | | **Aided Performance** | |
| --- | --- | --- | --- | --- |
|  | **Radiologists**  **AUC**  **(95% CI)** | **Non-Radiologist Physicians AUC**  **(95% CI)** | **Radiologists**  **AUC**  **(95% CI)** | **Non-Radiologist Physicians**  **AUC**  **(95% CI)** |
| Overall | 0.865  (0.856, 0.874) | 0.824  (0.819, 0.830) | 0.900  (0.893, 0.908) | 0.893  (0.889, 0.897) |
| Bones | 0.737  (0.688, 0.788) | 0.614  (0.584, 0.643) | 0.894  (0.862, 0.923) | 0.853  (0.833, 0.871) |
| Cardiac | 0.842  (0.817, 0.864) | 0.825  (0.811, 0.839) | 0.863  (0.838,0.884) | 0.873  (0.861, 0.885) |
| Hardware | 0.949  (0.936, 0.962) | 0.923  (0.914, 0.931) | 0.964  (0.953, 0.974) | 0.952  (0.944, 0.958) |
| Lungs | 0.890  (0.872, 0.906) | 0.813  (0.799, 0.827) | 0.919  (0.903, 0.934) | 0.904  (0.894, 0.915) |
| Mediastinum/Hila | 0.744  (0.711, 0.778) | 0.672  (0.651, 0.691) | 0.776  (0.748, 0.803) | 0.775  (0.760, 0.792) |
| Other | 0.699  (0.645, 0.752) | 0.664  (0.635, 0.696) | 0.807  (0.755, 0.849) | 0.799  (0.769, 0.825) |
| Pleura | 0.890  (0.869, 0.909) | 0.817  (0.803, 0.831) | 0.911  (0.892, 0.929) | 0.884  (0.872, 0.896) |
| Soft Tissues | 0.737  (0.651, 0.816) | 0.580  (0.532, 0.627) | 0.813  (0.733, 0.882) | 0.802  (0.759, 0.845) |

**Supplementary Table 4: Timing data of physician case read times when unaided and aided by the AI system.**

|  | **Unaided** | | **Aided** | |
| --- | --- | --- | --- | --- |
|  | **Median Time (s)** | **95% Bootstrap CI** | **Median Time (s)** | **95% Bootstrap CI** |
| **Radiologists** | 69.5 | [57, 77] | 67.5 | [61, 69] |
| **Non-Radiologist Physicians** | 107 | [86, 127] | 98.5 | [88, 103] |

**Supplementary Table 5: Individual physician performance unaided and aided by the AI system.**

| **Reader Specialty** | **Reader** | **Unaided AUC (95% Bootstrap CI)** | **Aided AUC (95% Bootstrap CI)** |
| --- | --- | --- | --- |
| Radiologist | R02 | 0.862 (0.845, 0.879) | 0.900 (0.886, 0.914) |
|  | R04 | 0.834 (0.818, 0.852) | 0.869 (0.856, 0.885) |
|  | R12 | 0.921 (0.910, 0.934) | 0.934 (0.923, 0.944) |
|  | R17 | 0.885 (0.871, 0.900) | 0.934 (0.924, 0.945) |
|  | R18 | 0.903 (0.889, 0.917) | 0.927 (0.915, 0.938) |
|  | R23 | 0.854 (0.838, 0.870) | 0.902 (0.890, 0.915) |
| Internal Medicine | R03 | 0.763 (0.743, 0.784) | 0.853 (0.836, 0.869) |
|  | R07 | 0.792 (0.773, 0.810) | 0.892 (0.879, 0.904) |
|  | R08 | 0.813 (0.795, 0.831) | 0.921 (0.908, 0.933) |
|  | R09 | 0.806 (0.788, 0.825) | 0.902 (0.889, 0.916) |
|  | R10 | 0.828 (0.813, 0.845) | 0.907 (0.895, 0.920) |
|  | R24 | 0.886 (0.873, 0.900) | 0.916 (0.904, 0.928) |
| Family Medicine | R06 | 0.872 (0.857, 0.888) | 0.912 (0.899, 0.925) |
|  | R11 | 0.795 (0.778, 0.814) | 0.881 (0.866, 0.895) |
|  | R13 | 0.831 (0.814, 0.849) | 0.899 (0.886, 0.912) |
|  | R14 | 0.835 (0.818, 0.852) | 0.876 (0.862, 0.891) |
|  | R21 | 0.784 (0.766, 0.801) | 0.928 (0.918, 0.939) |
|  | R22 | 0.877 (0.861, 0.892) | 0.932 (0.921, 0.943) |
| Emergency Medicine | R01 | 0.882 (0.868, 0.898) | 0.907 (0.895, 0.920) |
|  | R05 | 0.836 (0.819, 0.853) | 0.923 (0.911, 0.934) |
|  | R15 | 0.859 (0.843, 0.873) | 0.883 (0.869, 0.896) |
|  | R16 | 0.836 (0.821, 0.850) | 0.884 (0.873, 0.897) |
|  | R19 | 0.848 (0.832, 0.864) | 0.914 (0.902, 0.925) |
|  | R20 | 0.891 (0.880, 0.905) | 0.918 (0.907, 0.929) |

**Supplementary Table 6: Performance of the AI System on a subset of specific abnormalities.**

| **Category** | **Abnormality** | **n** | **Sensitivity** | **True Positive Cases** | **False Negative Cases** |
| --- | --- | --- | --- | --- | --- |
| Cardiac | Cardiomegaly | 314 | 0.892 | 280 | 34 |
| Mediastinum / Hila | Aortic Calcification | 197 | 0.909 | 179 | 18 |
| Mediastinum / Hila | Hilar enlargement | 252 | 0.849 | 214 | 38 |
| Mediastinum / Hila | Suspected thoracic aortic aneurysm | 35 | 0.914 | 32 | 3 |
| Lungs | Airspace opacity | 201 | 0.935 | 188 | 13 |
| Lungs | Vascular congestion / interstitial edema | 36 | 0.917 | 33 | 3 |
| Pleura | Pleural Effusion | 252 | 0.948 | 239 | 13 |
| Pleura | Pneumothorax | 204 | 0.912 | 186 | 18 |
| Bones | Fracture | 65 | 0.800 | 52 | 13 |
| Soft Tissues | Extensive subcutaneous emphysema | 71 | 1.000 | 71 | 0 |
| Hardware | Central venous catheter (CVC) | 47 | 0.979 | 46 | 1 |
| Hardware | Enteric tube | 20 | 1.000 | 20 | 0 |
| Other | Carotid atherosclerosis | 57 | 0.930 | 53 | 4 |
| Other | Potential bowel obstruction | 45 | 1.000 | 45 | 0 |
| Other | Peripheral vascular disease | 34 | 0.735 | 25 | 9 |
| Other | Abnormal stomach distention | 25 | 0.880 | 22 | 3 |
